# Clinical outcomes of women with ovarian metastases of colorectal cancer treated with oophorectomy with respect to their somatic mutation profiles

**DOI:** 10.18632/oncotarget.24735

**Published:** 2018-03-27

**Authors:** Yoshiko Mori, Akihiro Nyuya, Kazuya Yasui, Toshiaki Toshima, Takashi Kawai, Fumitaka Taniguchi, Keisuke Kimura, Ryo Inada, Masahiko Nishizaki, Junko Haraga, Keiichiro Nakamura, Yuzo Umeda, Hiroyuki Kishimoto, Toshiyoshi Fujiwara, Yosuke Katata, Yoshiyuki Yamaguchi, Takeshi Nagasaka

**Affiliations:** ^1^ Departments of Gastroenterological Surgery, Okayama University Graduate School of Medicine Dentistry and Pharmaceutical Sciences, Okayama, Japan; ^2^ Clinical Genomic Medicine, Okayama University Graduate School of Medicine Dentistry and Pharmaceutical Sciences, Okayama, Japan; ^3^ Department of Clinical Oncology, Kawasaki Medical School, Kurashiki City, Japan; ^4^ Obstetrics and Gynecology, Okayama University Graduate School of Medicine Dentistry and Pharmaceutical Sciences, Okayama, Japan

**Keywords:** ovarian metastases, colorectal cancer, ovarian metastatectomy, BRAF, RAS

## Abstract

We clarified the clinical prevalence of ovarian metastases from colorectal cancers (CRCs) in 296 female patients with CRC and evaluated clinical outcomes with relation to their mutational profiles, such as BRAF/KRAS mutation and microsatellite instability (MSI) status. The female CRCs were categorised into three subsets: CRCs with ovarian metastases [6.4% (*n =* 19), 5-year overall survival (OS) = 24.7%], CRCs with extra-ovarian metastases only [32.4% (*n =* 96), 5-year OS = 34.5%] and CRCs without any recurrence or metastasis [61.2% (*n =* 181), 5-year OS = 91.3%]. All patients with ovarian metastases underwent oophorectomy; of these, 9 who received preoperative chemotherapy had measurable metastases to extra-ovarian sites and the ovaries. Although 5 of 9 (56%) achieved partial response or complete response at extra-ovarian sites, no patient archived objective response at ovarian sites. Regarding the mutation profiles, in CRCs with extra-ovarian metastases only, the median survival time (MST) after initial treatments to progression to stage IV or recurrence was 13 [95% confidence interval (CI): 7–16 months] in BRAF-mutant and 34 months (95% CI: 22–58 months) in BRAF wild-type (*P =* 0.0033). Although ovarian metastases demonstrated poor response to systemic chemotherapy in CRCs with ovarian metastases, the MST after initial treatments to progression to stage IV or recurrence was 22 (95% CI: 21–25 months) in BRAF-mutant and 38 months (95% CI: 24–42 months) in BRAF wild-type (*P =* 0.0398). The outcomes of patients with ovarian metastases could be improved by oophorectomy regardless of their mutation profiles.

## INTRODUCTION

Ovarian metastasis is estimated to account for 5%–30% of all ovarian malignancies [1–5] and most frequently originates from colorectal cancers (CRC), followed by cancers of the endometrium, stomach, appendix and breast [6]. Although 12.5%–49% of metastatic ovarian cancers originate from CRCs, the ovary is an uncommon site for metastasis from advanced CRCs. In fact, synchronous and metachronous ovarian metastases were reported in approximately 9% and 7% of women with CRCs, respectively [7–11].

In the past decade, remarkable progress has been achieved in the treatment of advanced CRC, particularly after the introduction of effective systemic chemotherapeutic regimens, including oxaliplatin and irinotecan, and molecular-targeted antibodies [12]. These novel agents have enabled better tumour response rates and overall survivals (OS) in patients. However, despite these significant improvements, patients with ovarian metastases from CRCs have a worse prognosis than patients with CRCs that metastasised to other sites [13].

It remains unknown whether the adverse prognosis of ovarian metastases relative to the metastases to other sites reflects a more advanced stage of the disease, intrinsic aggressiveness of the disease, or reduced sensitivity to systemic chemotherapy [14]. A recent study on patients with CRCs demonstrated that the responses to chemotherapy were less favourable in those with ovarian metastases than in those with extra-ovarian metastases [13]. Another study reported that the tumour response rate after fluorouracil-based chemotherapy was 40% in patients with extra-ovarian metastases and only 5% in those with ovarian metastases [14]. The absence of objective tumour responses in 22 patients with CRCs who had ovarian metastases and the tumour control rate of 65% in patients with extra-ovarian metastatic lesions supported these earlier results [13]. Lee et al. reported that patients with CRCs who underwent oophorectomy or metastatectomy for ovarian metastases before undergoing chemotherapy survived significantly longer than those who did not undergo oophorectomy (28.1 vs. 21.2 months) [15]. In the multivariate analysis, the absence of oophorectomy was an independent prognostic factor for worse survival and had a relative risk of 1.954. In summary, the resection of ovarian metastases seemed to confer survival benefits on CRC patients.

Recently, treatment strategies for advanced CRC have leaned towards mutation profile-based precision medicine; in other words, the mutational profiles of primary tumours and metastatic lesions are important when determining treatment strategies [16–25]. However, to the best of our knowledge, no study has evaluated the clinical outcomes of patients with ovarian metastases from CRCs according to their *BRAF* (v-Raf murine sarcoma viral oncogene homolog B) *or KRAS* (V-Ki-ras2 Kirsten rat sarcoma viral oncogene homolog) mutation profiles and status of microsatellite instability (MSI).

In this study, we first established the clinical prevalence of ovarian metastases from CRC from a cohort of 666 patients. Next, we analysed the clinical outcomes and treatment strategies, according to the mutational profiles of the CRCs. Finally, we evaluated the clinicopathologic features of women with ovarian metastases from CRC and attempted to determine the efficacy of oophorectomy in such cases.

## RESULTS

### Clinicopathologic features of ovarian metastases in women with CRC

**Figure 1:**
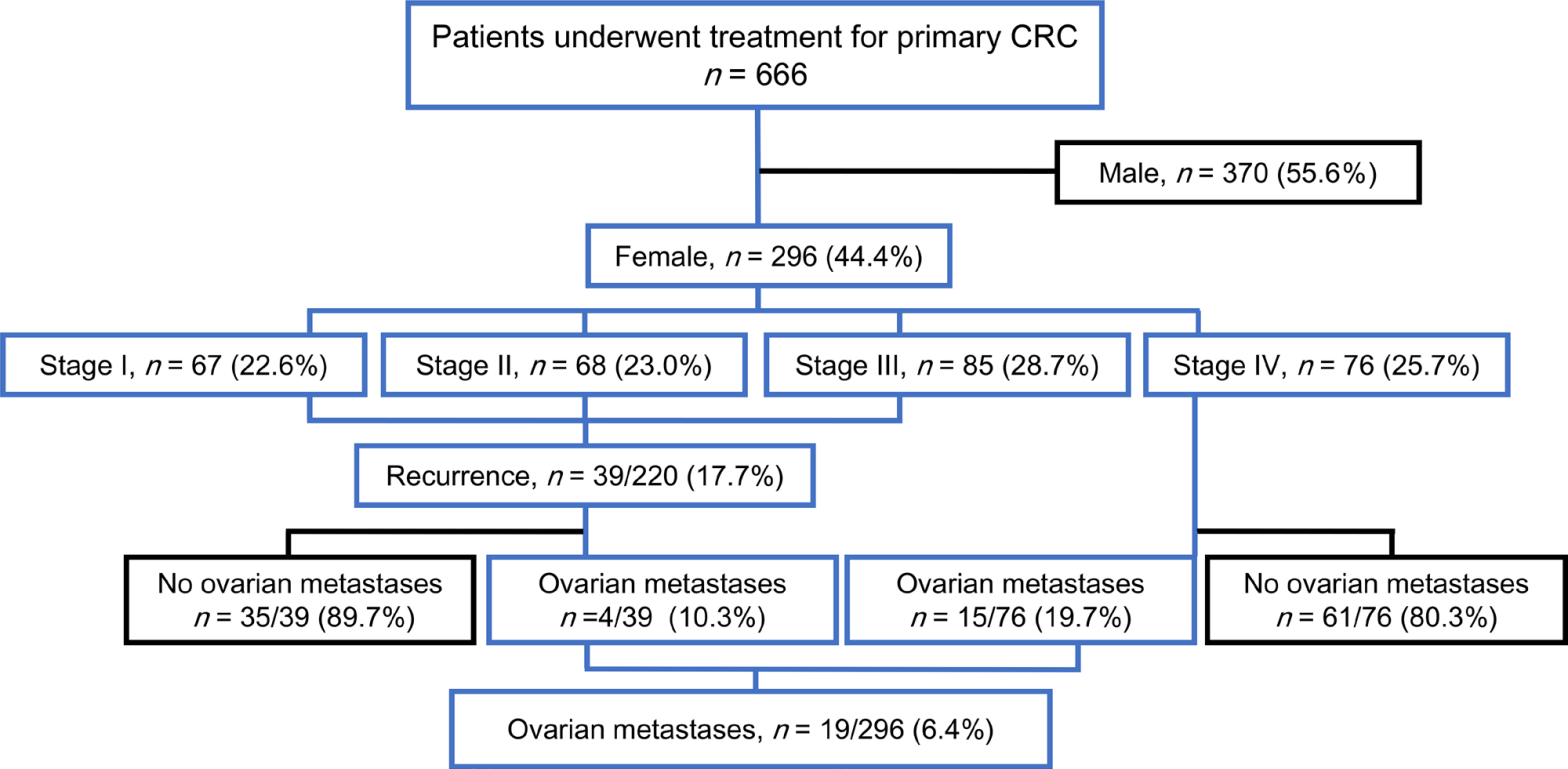
The STROBE diagram of the patient cohort

We first evaluated the clinical prevalence of ovarian metastases from CRCs in 296 female patients in our cohort (Figure 1). These cases were categorised into three subsets: those with ovarian metastases (*n =* 19, 6.4%), those with extra-ovarian metastases only (*n =* 96, 32.4%) and those without recurrence or metastasis (*n =* 181, 61.1%) (Table 1). Patients who developed ovarian metastases were younger than those who developed extra-ovarian metastases only (*P* < 0.0001) and had a median age of 51 years (range, 34–80) upon diagnosis of the first ovarian metastasis. Nine of the 19 women (47.4%) with ovarian metastases were diagnosed at ages younger than 50 years. With respect to the location of the primary colorectal tumours, 18 (94.7%) of the patients with ovarian metastases showed the presence of tumours in the colon and only 1 case showed a primary rectal tumour.

**Table 1:** Characteristics of patients with and without ovarian metastasis

Demographics		Total	With ovarian metastasis	With extraovarian metastases only	No recurrence nor metastasis	*P* value
		(*n =* 296)	(*n =* 19)	(*n =* 96)	(*n =* 181)	
Age	over 65 years	160 (54.1)	2 (10.5)	50 (52.1)	108 (59.7)	0.0009^a^
	65 years or less	136 (45.9)	17 (89.5)	46 (47.9)	73 (40.3)	0.0002^b^
Location of primary tumour	right colon	120 (40.5)	9 (47.4)	33 (34.4)	78 (43.1)	0.0284^c^
	left colon	111 (37.5)	9 (47.4)	35 (36.5)	67 (37.0)	0.0397^d^
	rectum	65 (22.0)	1 (5.3)	28 (29.2)	36 (19.9)	
UICC stage	I	67 (22.6)	0 (0.0)	3 (3.1)	64 (35.4)	0.416^a^
	II	69 (23.3)	0 (0.0)	8 (8.3)	60 (33.2)	< 0.0001^b^
	III	84 (28.4)	4 (21.1)	24 (25.0)	57 (31.5)	
	IV	76 (25.7)	15 (79.0)	61 (63.5)	0 (0.0)	
T factor	T0–T3	233 (79.3)	9 (52.9)	54 (56.3)	170 (93.9)	0.801^a^
	T4	61 (20.8)	8 (47.1)	42 (43.8)	11 (6.1)	< 0.0001^b^
	Tx	2	2	0	0	
Histology	well or mod	270 (91.2)	17 (89.5)	81 (84.4)	172 (95.0)	0.567^a^
	poor or muc	26 (8.8)	2 (10.5)	15 (15.6)	9 (5.0)	0.014^b^
*KRAS/BRAF* mutation status in primary lesions	*KRAS*-mt	106 (35.8)	5 (26.3)	35 (36.5)	66 (36.5)	0.314^a^
	*BRAF*-mt	23 (7.8)	3 (15.8)	6 (6.3)	14 (7.7)	0.662^b^
	Wild-type of the both genes	167 (56.4)	11 (57.9)	55 (57.3)	101 (55.8)	
MSI status in primary lesions	MSI	21 (7.1)	0 (0.0)	6 (6.3)	15 (8.3)	0.263^a^
	Non-MSI	275 (92.9)	19 (100.0)	90 (93.8)	166 (91.7)	0.378^b^

^*^well or mod; well or moderately differentiated adenocarcinoma, poor or muc; poorly differentiated or mucinous adenocarcinoma, MSI; microsatellite instability, ^a^*P* value; calculated by chi-square test between ovarian metastasis group and extraovarian metastasis only group, ^b^*P* value; calculated by chi-square test among ovarian metastasis group, extraovarian metastasis only group and no recurrence nor metastasis group, ^c^*P* value; calculated by chi-square test of colon (including right and left colon) versus rectum between ovarian metastasis group and extraovarian metastasis only group, ^d^*P* value; calculated by chi-square test of colon (including right and left colon) versus rectum among ovarian metastasis group, extraovarian metastasis only group and no recurrence nor metastasis group.

### Genetic profiles of patients with CRCs who had ovarian metastases

As shown in Table 1, the *KRAS* mutation in primary tumour was equal in frequency of occurrence among the subsets of patients with CRC and affected 5 of 19 patients (26.3%) with ovarian metastases, 35 of 96 patients (36.5%) with extra-ovarian metastases only and 66 of 181 patients (36.5%) without any recurrence or metastases. In the analysis of the *BRAF* exon 15 mutation in primary tumour site, 23 of the 296 female patients with CRC (7.8%) showed the presence of the *BRAF* mutation and all of them carried the V600E mutation. The *BRAF* V600E mutation had a non-significant tendency to be more frequent in patients with ovarian metastases (3 of 19, 15.8%) than in those with either extra-ovarian metastases only (6 of 96, 6.3%), or without any recurrence or metastasis (14 of 181, 7.7%). The MSI phenotype was negative in all patients with ovarian metastases but was observed to be equal between those with extra-ovarian metastases only (6 of 96, 6.3%) and those without any recurrence or metastasis (15 of 181, 8.3%).

### Clinical outcomes of women with CRC

**Figure 2:**
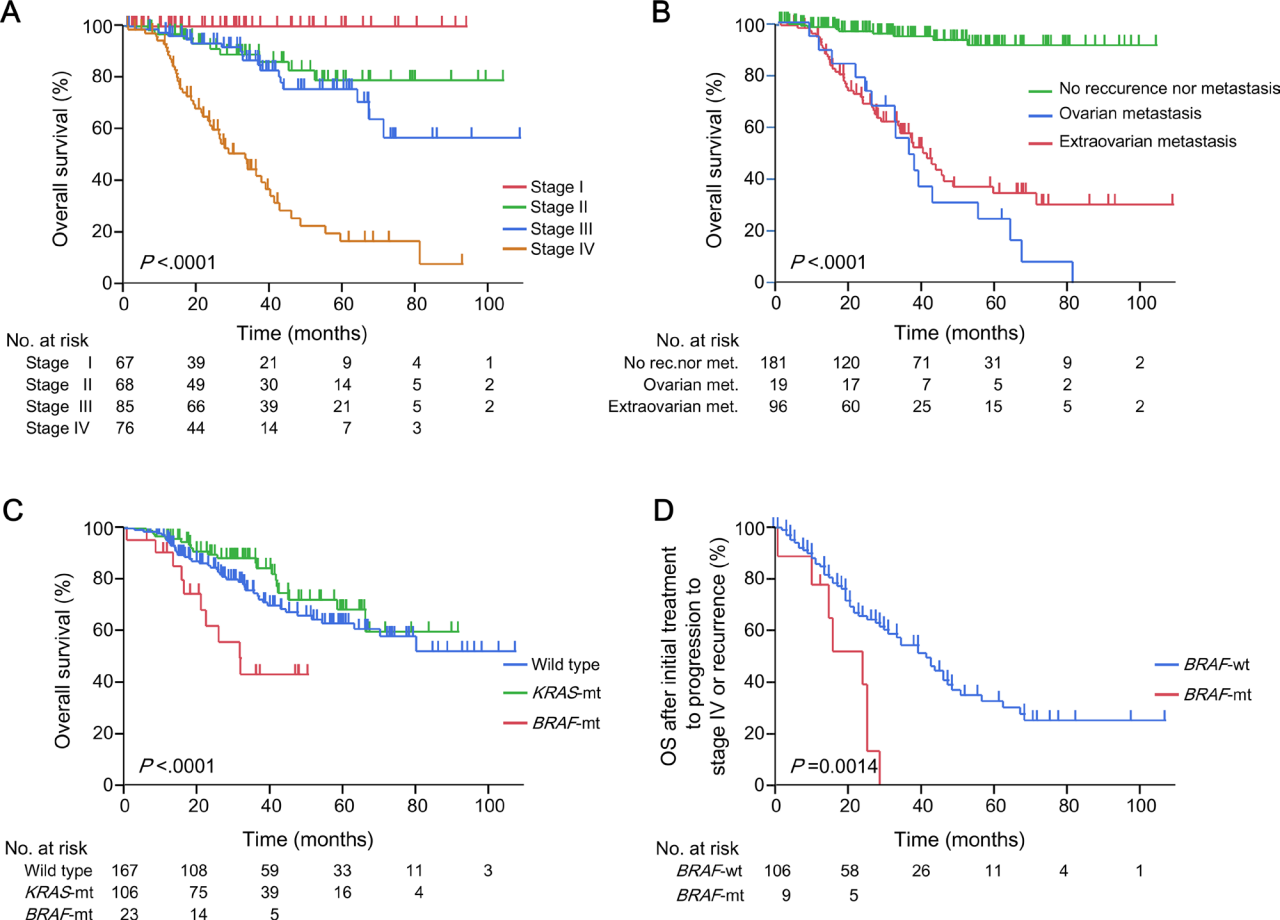
Clinical outcomes of 296 women with CRCs Kaplan–Meier estimates according to (**A**) UICC stage; (**B**) status of ovarian and other metastases; and (**C**) the *BRAF or KRAS* mutation status, in 296 women with CRCs. (**D)** Kaplan–Meier estimates according to the *BRAF or KRAS* mutation status in 115 women with CRCs who had metachronous or synchronous metastases. *P* values were calculated using the log-rank test. CRC, colorectal cancer; UICC, Union for International Cancer Control; Wild type, wild type of both *KRAS* and *BRAF* genes; *KRAS*-mt, *KRAS* mutations; *BRAF*-mt, *BRAF* V600E mutation; *BRAF*-wt, *BRAF* wild type (*KRAS* mutations and wild type of both genes).

The median follow-up period of the 296 women with CRC was 30 months (range, 0–108). The outcomes of these patients according to their age, primary tumour location, histology and MSI status are summarised in Supplementary Figure 1. The 5-year OS rates were 100%, 79.3%, 76.0% and 17.6% for women with CRCs at Union for International Cancer Control (UICC) stages I, II, III and IV, respectively (*P* < 0.0001, Figure 2A), and 24.7%, 34.5% and 91.3% for those with ovarian metastases, extra-ovarian metastases only and no recurrence or metastasis, respectively (*P* < 0.0001, Figure 2B). The 3-year OS rates differed significantly among those with the *BRAF* V600E mutation, *KRAS* mutations and wild-type genes (43.6%, 86.5% and 73.3%, respectively; *P* < 0.0001, Figure 2C). Among the 115 women who had synchronous or metachronous metastases of CRC (76 patients were at stage IV at diagnosis and 39 patients experienced recurrences after curative surgery), the median survival times after initial treatment to progression to stage IV or recurrence were 21 months [95% confidence interval (CI), 1–22 months] in patients with the *BRAF* mutation and 36 months (95% CI, 26–42 months) in those without the *BRAF* mutation (*BRAF* wild type) (*P =* 0.0014, Figure 2D).

Table 2 shows the univariate and multivariate analyses of OS rates. In the univariate and multivariate analysis, the UICC stage, histology and the status of ovarian metastasis and *BRAF* or *KRAS* mutations were significantly associated with the OS.

**Table 2:** Univariate and Multivariate analysis for survival

Clinicopathologic Factor	OS			
	Univariate		Multivariate	
	RR (95% CI)	*P* value	RR (95% CI)	*P* value
Age		0.8626		0.0364
> 65 (vs < 65)	0.96 (0.60–1.53)		1.74 (1.04–2.94)	
Location of primary tumour		0.8633		0.9198
right colon (vs left colon and rectum)	0.96 (0.59–1.53)		1.03 (0.58–1.81)	
UICC stage		**< 0.0001**		**0.0006**
II (vs I)	1.659 (3.07–∞)	0.0017	9.42e+8 (1.52–∞)	0.0188
III (vs I)	2.40e+9 (4.70–∞)	0.0001	1.18e+9 (1.97–∞)	0.0083
IV (vs I)	1.19e+10 (1.48e+6–∞)	<.0001	2.63e+9 (4.30–∞)	0.0003
III (vs II)	1.45 (0.66–3.40)	0.3614	1.25 (0.54–3.10)	0.6081
IV (vs II)	7.16 (3.66–15.7)	< 0.0001	2.79 (1.22–7.12)	0.0141
IV (vs III)	4.94 (2.87–8.92)	< 0.0001	2.23 (1.23–4.29)	0.0080
Histology		**0.0004**		**0.0145**
poor or muc (vs well or mod)	3.53 (1.84–6.28)		2.50 (1.21–4.82)	
Status of ovarian metastasis		**< 0.0001**		**< 0. 0001**
with ovarian metastasis (vs no recurrence nor metastasis)	15.8 (7.11–37.3)	< 0.0001	7.79 (2.80–22.4)	0.0001
with extraovarian metastases only (vs no recurrence nor metastasis)	11.1 (5.70–24.2)	< 0.0001	6.79 (2.94–16.7)	< 0.0001
with ovarian metastasis (with extraovarian metastases only)	1.42 (0.78–2.46)	0.2371	1.15 (0.58–2.18)	0.6841
MSI status in primary lesions		0.8119		0.3993
MSI (vs non-MSI)	1.12 (0.39–2.51)		1.56 (0.51–3.84)	
*BRAF/KRAS* mutational status in primary lesions		**0.0130**		**0.0087**
*BRAF*-mt (vs wild-type of both genes)	2.46 (1.16–4.74)	0.0212	2.37 (1.00–9.61)	0.0030
*KRAS*-mt (vs wild-type of both genes)	0.70 (0.40–1.19)	0.1923	0.58 (0.30–1.06)	0.0782
*BRAF*-mt (vs *KRAS*-mt)	3.50 (1.56–7.40)	0.0034	4.09 (1.65–9.61)	0.0030

^*^well or mod; well or moderately differentiated adenocarcinoma, poor or muc; poorly differentiated or mucinous adenocarcinoma, MSI; microsatellite instability.

### Mutation status concordance between primary colorectal tumour lesions and ovarian metastatic sites

Supplementary Table 1 presents the analysis of the clinicopathological factors, including the mutation status concordance of 19 women with ovarian metastasis from CRC who underwent hemi-oophorectomy (*n =* 8) and bilateral oophorectomy (*n =* 11). Of these 19 patients, the mutation spectrum of 18 patients could be analysed in their primary tumour lesions and corresponding ovarian metastatic sites. All 18 patients analyzed presented the *BRAF* mutation and MSI status concordance. In contrast, a disconcordance of the *KARS* mutation status was determined in 16.7% (3 of 18) of paired primary tumours and corresponding ovarian metastases. In addition, the 2 cases with *KRAS* wild type in primary tumours demonstrated *KRAS* G12S or G12V in the corresponding ovarian metastasis. The remaining case presented *KRAS* G12D in the primary tumour but none in ovarian metastasis.

### Responses to preoperative chemotherapy in patients with CRCs who had ovarian metastases

**Figure 3:**
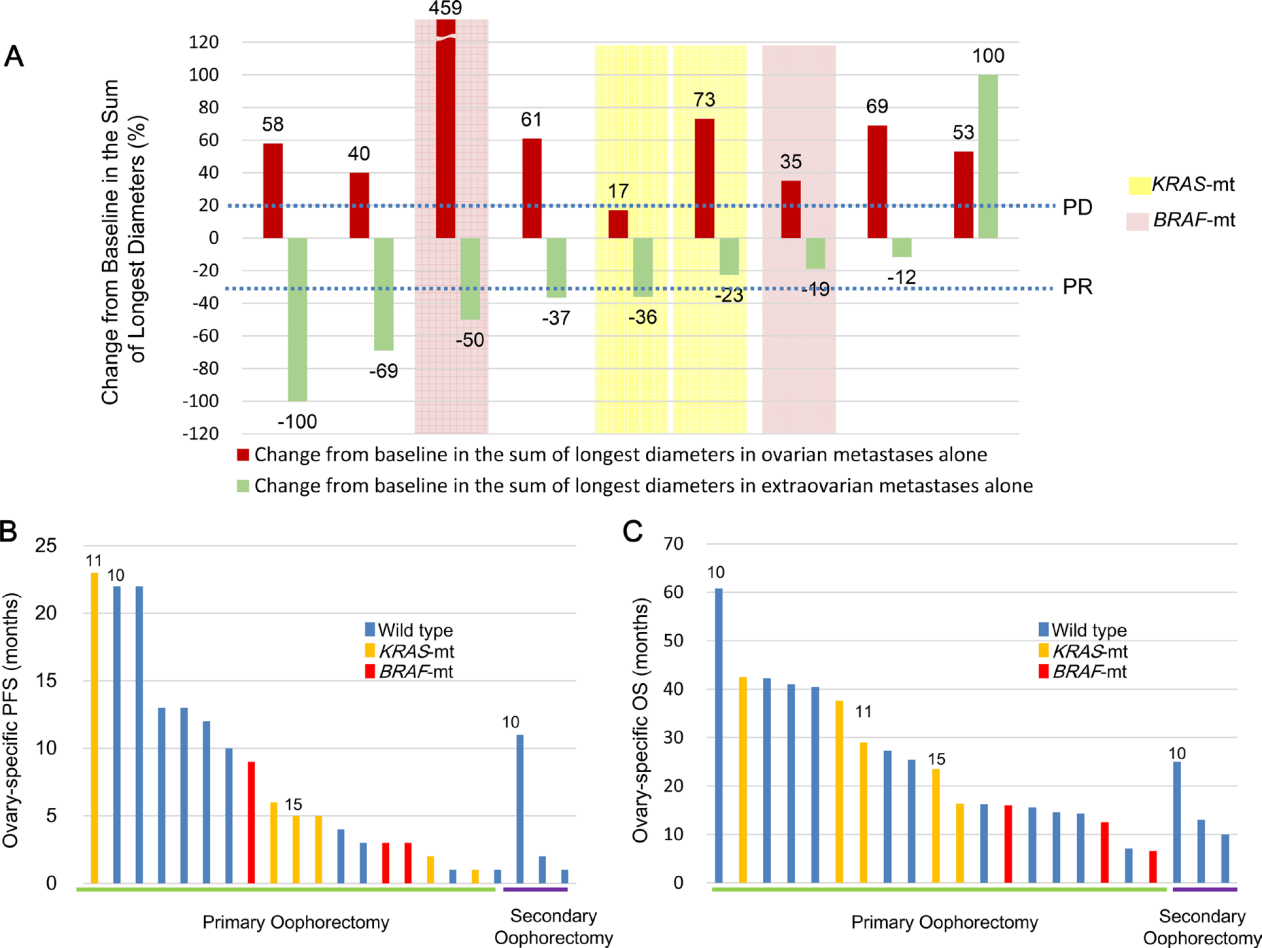
Responses to systemic chemotherapy and outcomes after undergoing oophorectomy (**A**) Responses to systemic chemotherapy in 9 women with CRCs that had metastasised to the ovary or to other organs, or both; (**B**) Ovarian-specific PFS; and (**C**) OS after oophorectomy. Numbers indicate the number of patients (Supplementary Table 1) who were censored (i.e., alive at the time of data extraction). CRC, colorectal cancer; PFS, progression-free survival; OS, overall survival; Wild type, wild type of both *KRAS* and *BRAF* genes in the ovarian metastatic lesions; *KRAS*-mt, *KRAS* mutations in the ovarian metastatic lesions; *BRAF*-mt, *BRAF* V600E mutation in the ovarian metastatic lesions.

The response to preoperative chemotherapy was evaluated in 10 of 19 patients with ovarian and extra-ovarian metastases. Nearly all patients with ovarian metastases were unresponsive to preoperative chemotherapy, but those with extra-ovarian metastases had good responses (Figure 3A and Supplementary Table 2). Of the 10 patients who received preoperative chemotherapy, 9 had measurable metastases to extra-ovarian sites and to the ovaries; 5 of these 9 patients (56%) achieved partial response (PR) or complete response (CR) at the extra-ovarian sites. On the other hand, only 1 of the 9 patients (11%) achieved stable disease (SD) at ovarian sites, whereas 8 of the 9 patients (89%) exhibited progressive disease (PD).

### Survival and timing of oophorectomy for patients with CRCs who had ovarian metastases

In the patients with CRCs who had ovarian metastases, the ovarian-specific median survival time (MST) was 25 months (95% CI, 15–40 months) (Supplementary Figure 2A), whereas the median ovarian-specific progression-free survival (PFS) was 5 months (95% CI, 3–12 months) (Supplementary Figure 2B).

**Figure 4:**
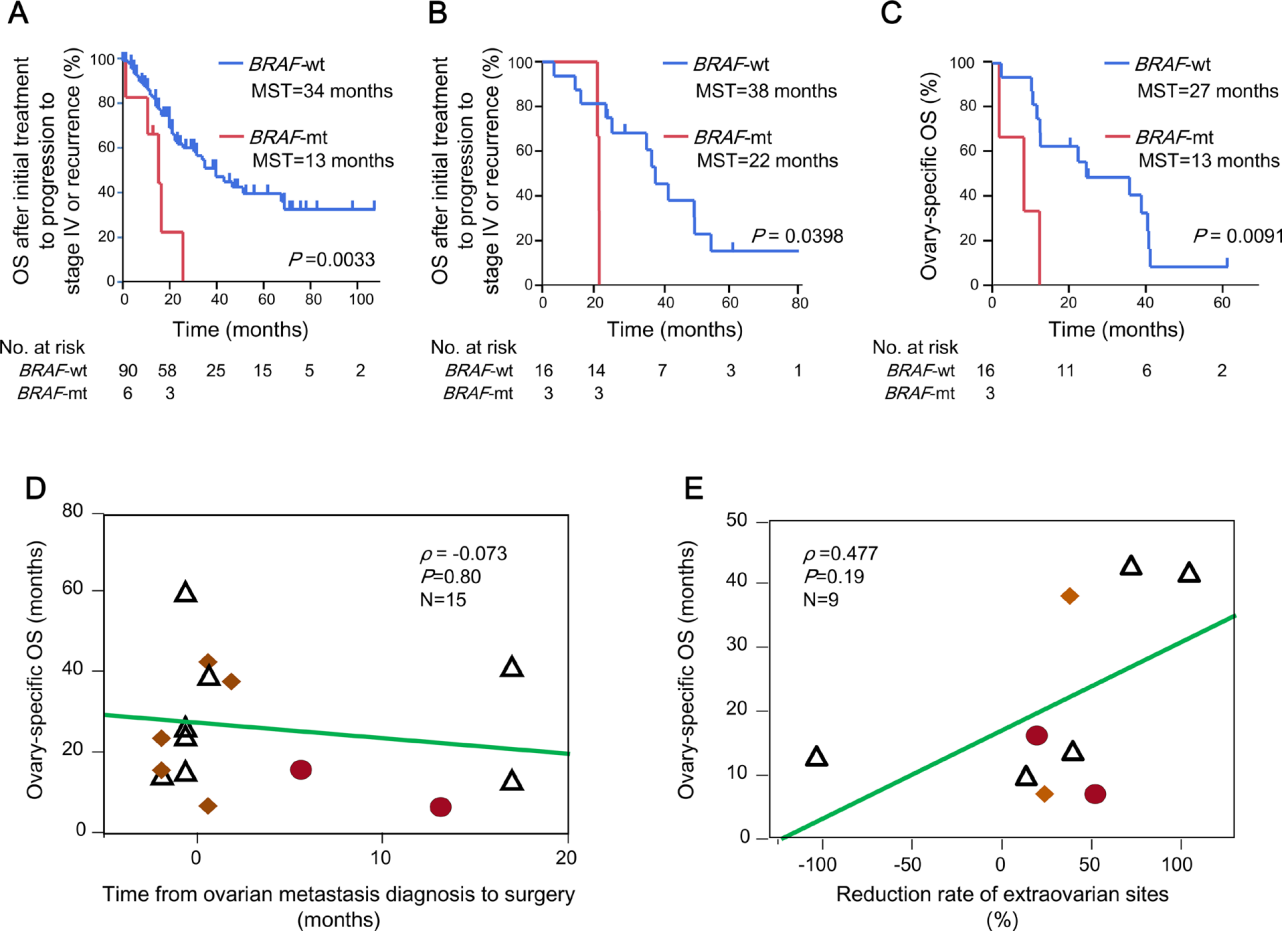
Clinical features of the female patients with ovarian metastases from CRC Kaplan–Meier estimates of the OS after initial treatment to progression to stage IV or recurrence for (**A**) 96 women with CRCs who had extra-ovarian metastases only, and (**B**) 19 women with ovarian metastases according to their *BRAF* mutation status. (**C**) Kaplan–Meier curves of the ovarian-specific OS for 19 women with ovarian metastases, according to their *BRAF* mutation status. MST denotes the median duration of the OS after initial treatment to progression to stage IV or recurrence and ovarian-specific OS. *P* values were calculated using the log-rank test. (**D**) The relationship between the ovarian-specific OS and the interval between the diagnosis of ovarian metastasis and the initial oophorectomy. (**E**) The relationship between the ovarian-specific OS and the rates of reduction of extra-ovarian metastases in 9 women treated with systemic chemotherapy. Among the 19 patients with ovarian metastases, 10 were treated with chemotherapy before oophorectomy; of these, 9 (90%) had measurable metastases to the ovaries and extra-ovarian sites. *ρ* denotes the Spearman’s rank correlation coefficient. Red circles denote patients with the *BRAF* V600E mutation in the ovarian metastatic lesions. Yellow squares denote patients with *KRAS* mutations in the ovarian metastatic lesions. CRC, colorectal cancer; OS, overall survival; MST, median survival time; *BRAF*-mt, *BRAF* V600E mutation; *BRAF*-wt, *BRAF* wild type (*KRAS* mutations and wild type of the both genes).

Because women with CRCs who had the *BRAF* V600E mutation had a worse prognosis than those with the *BRAF* wild-type (*KRAS* mutation and the wild type of both genes) gene (Figure 2A and 2B), we established the clinical features of the 115 women with metachronous or synchronous metastases, including ovarian metastases from CRC, according to the *BRAF* mutation status. In the 96 patients with only extra-ovarian metastases, the MST after initial treatment to progression to stage IV or recurrence was 13 months (95% CI, 1–22 months) for patients with the *BRAF* mutation and 34 months (95% CI, 22–58 months) for patients without the *BRAF* mutation (*P* = 0.0033, Figure 4A).

Of 19 patients with ovarian metastases, 15 were diagnosed at stage IV and 4 developed recurrences after undergoing curative resections of primary colorectal tumours. Of 4 patients who developed metachronous ovarian metastases, 2 had the *BRAF* V600E mutation, 1 had the *KRAS* mutation and 1 had the wild types of both genes in the primary tumour (the patients who had the wild types of both genes in the primary tumour demonstrated the *KRAS* mutation in the ovarian metastasis). The OS after initial treatments for recurrences was 22 and 24 months, respectively, in 2 patients with *BRAF* mutants, 50 months in 1 patient with the *KRAS* mutation and 50 months in 1 patient with wild types of both genes in the primary tumour (50 months in the 2 patients with the *KRAS* mutation in the ovarian metastases). In patients with ovarian metastases, the MSTs after initial treatments to progression to stage IV or recurrence were 22 (95% CI: 21–25 months) and 38 months (95% CI: 24–42 months) in those with and without the *BRAF* mutation, respectively (*P* = 0.0398; Figure 4B). The MST of the ovarian-specific OS was 13 (95% CI: 7–16 months) and 27 months (95% CI: 16–41 months) in patients with the *BRAF* V600E mutation and the *BRAF* wild-type gene, respectively (*P* = 0.0091; Figure 4C).

As expected, the success rates of the first oophorectomies significantly affected survivals. Those with a curability rate of R0 or R1 had an MST of the ovarian-specific OS of 43 months (95% CI, 16 months–not calculated), whereas those with R2 disease had an MST of 16 months (95% CI, 13–38 months) (*P* = 0.0058, Supplementary Figure 2C). In contrast, no relationship was observed between the ovarian-specific OS from the first oophorectomy and the interval from the diagnosis of ovarian metastasis to oophorectomy (*ρ* = −0.073, Figure 4D). Similarly, the ovarian-specific OS after the initial oophorectomies exhibited a weakly positive, but non-significant, association with the reduction rates of extra-ovarian sites (*ρ* = 0.477, Figure 4E).

Notably, the additional knowledge gained from the post-oophorectomy pathologic examinations suggests that the possibility of another occult cancer of the ovary should be approached with caution. Of the 11 cases that underwent bilateral oophorectomies, 9 appeared to have either unilateral or no metastases at the time of clinical and surgical diagnoses; however, 5 of these 9 cases (56%) were eventually diagnosed with bilateral metastases.

## DISCUSSION

The growth of ovarian tumours can cause serious clinical symptoms, such as abdominal pain, a heavy feeling in the abdomen, constipation, frequent urination and anorexia. Systemic therapy does not usually improve these symptoms, even with the availability of new molecular-targeted agents or cytotoxic chemotherapy regimens [13–15]. In this retrospective study, we determined the clinical prevalence, outcomes and appropriate treatment strategies in a large cohort of patients with ovarian metastases from CRCs, according to their somatic mutation profiles.

Previous studies have demonstrated that female patients with CRC who developed ovarian metastases were younger, and more than 80% of the primary tumours were in the colon [9, 15]. Consistent with previous reports, in our study, 18 (95%) of the 19 primary tumours from patients with ovarian metastases were in the colon. This trend strongly suggested that ovarian metastases from CRCs spread via dissemination rather than by haematogenous or lymphogenous routes. However, in accordance with a previous study [9], there was no association between the depths of the invasion (T factor) and the frequency of ovarian metastases.

While the concordant *BRAF* mutation and the MSI status were observed in 100% (18 of 18) paired samples of primary colorectal tumours and corresponding ovarian metastases, the *KRAS* mutation concordance was observed in 83.3% (15 of 18) of them. Baas et al. demonstrated the overall concordance rate of 93% (range, 76%–100%) for the *KRAS* mutation status between primary colorectal tumours and corresponding metastatic sites by summarising 21 studies that reported concordance of the *KRAS* mutation status [26]. The two cases who showed the primary tumours with *KRAS* wild type and the corresponding ovarian metastasis with *KRAS* G12S or G12V did not received any anti-EGFR antibody before oophorectomies. This strongly suggests that such disconcordance does not result from newly acquired mutations obtained from chemotherapies with anti-EGFR antibody.

The results of this study revealed that the responses of patients with ovarian metastases were worse than those with extra-ovarian metastases (0% vs. 56%). Similarly, among 20 evaluable patients, Goere et al. reported that responses to cytotoxic chemotherapy were worse in patients with ovarian metastasis than in those with extra-ovarian lesions (0% vs. 35%) [13]. Among 33 patients who did not undergo oophorectomies, Lee et al. reported that responses to chemotherapy in those with ovarian and extra-ovarian metastases were 18.2% and 33.3%, respectively [15].

In contrast to the poor responses to chemotherapy in CRC patients with ovarian metastases, there was an ovarian-specific survival benefit in patients who underwent oophorectomies, with ovarian-specific MSTs of 43 and 16 months for patients who underwent complete resections (R0 or 1) and palliative debulking (R2), respectively. Notably, even palliative debulking prolonged the durations of the absence of either serious abdominal symptoms or anorexia due to ovarian metastases. We did not analyse the patients who did not undergo oophorectomies; however, in an earlier study, Lee et al. analysed 130 patients with ovarian metastases from CRCs and found that the MSTs after the diagnoses of ovarian metastasis were 20.8 months in 83 patients who underwent oophorectomy and 10.9 months in 47 patients who did not [15]. Therefore, our study and previous reports collectively suggested that although oophorectomy remains an uncommon perioperative treatment procedure, it is a promising and feasible management option for patients with CRCs who have ovarian metastases. Thus, ovarian metastasis is rather close to dissemination than a metastasis, oophorectomies play an important role for local control for the disease. Surgery will have some benefit for limited peritoneal dissemination in patients with CRC. Conversely, chemotherapy will have limited benefit for peritoneal dissemination.

Based on recent studies that showed worse prognoses in patients with advanced CRCs who harboured the *BRAF* V600E mutation than those who did not [16–19, 22, 23, 27], we analysed the genetic backgrounds and prognoses of female patients with CRCs in our cohort. Expectedly, patients with the *BRAF* V600E mutation demonstrated the worst OS among all female patients with different stages of CRC. This trend was also observed for the OS from initial treatment to progression to stage IV or recurrence and the ovarian-specific OS among female patients with CRC who had ovarian metastases as well as among those with extra-ovarian metastases only. Furthermore, 2 of 4 patients who developed ovarian metastases after undergoing curative resections of primary tumours presented the *BRAF* V600E mutation in primary tumours. Randomised phase III trials on first-line chemotherapy demonstrated that the OS in patients with the *BRAF* mutation ranged between 9 and 15 months [18, 22, 28, 29]. In this study, the MST after the initial treatment to stage IV or recurrence was 13 months in *BRAF*-mutant patients with CRC who had extra-ovarian metastases only; however, the MST after the initial treatment to stage IV or recurrence reached 22 months in *BRAF*-mutant patients with ovarian metastases.

As our study and previous studies demonstrated [13–15], resistance of ovarian metastases to chemotherapy was a common feature of ovarian metastases from CRC. Indeed, although patients with ovarian metastases had relatively the higher proportion for *BRAF* mutation compared with patients without ovarian metastases, patients with *BRAF* mutation had the poor survival irrespective of type of metastases. Thus *BRAF/KRAS* mutation or MSI status could not explain the reason why chemotherapy gives benefit so small to ovarian metastasis even though it has objective response to extra-ovarian metastasis.

Of 19 patients, the *BRAF* mutation was observed in 3 patients with variable duration time (0, 5.5 and 12.0 months) to initial oophorectomy from the diagnosis of ovarian metastasis and survival times after initial oophorectomy was relatively shorter than those observed in patients with *BRAF* wild-type (Figure 4D). In contrast to the survival time, responses to systemic chemotherapy in extra-ovarian metastatic sites observed in 2 *BRAF*-mutant patients (response rates, 19% and 50%) were average; however, oophorectomy-specific OSs were relatively shorter in them than in patients with *BRAF* wild-type (Figure 4E). From these results, it can be inferred that colorectal tumours with the *BRAF* mutation can shrink visibly but the PFS was shorter during systemic chemotherapy.

This study had several limitations: retrospective design, failure to include patients with ovarian metastases who did not undergo oophorectomy and a single-centre cohort. However, this study confirmed several essential findings regarding ovarian metastases of CRC. First, in corroboration with previous reports, the clinical prevalence of ovarian metastases from CRC was approximately 7%. Second, women with advanced CRCs with *BRAF* mutations demonstrated poor prognoses despite the status of ovarian metastasis, and the occurrence of the *BRAF* mutation was higher in female patients with CRCs who had ovarian metastases than those who did not. Third, ovarian metastases from CRCs did not respond to any systemic chemotherapy despite responses from other metastatic sites. Therefore, irrespective of their somatic mutation profiles, ovarian metastatectomy at the time of achieving the disease control at extra-ovarian metastases will enhance the outcome of women with colorectal ovarian metastases.

## MATERIALS AND METHODS

### Patients

We retrospectively analysed a cohort of 666 consecutive patients with CRC who underwent surgical resections at the Okayama University Hospital from January 2007 to September 2015. This cohort included 296 female patients; 20 of these were suspected to have ovarian metastases from CRCs and had undergone one or two oophorectomy procedures. In our department, all such patients who were suspected to have ovarian metastases from CRC, based on their radiologic evaluations, are eligible for oophorectomy. Therefore, in this study, we could not include patients with ovarian metastases who did not undergo oophorectomy. After careful examinations of the surgical and pathologic records and images, we excluded one patient who underwent oophorectomy for a direct ovarian invasion from the peritoneal metastasis of primary CRC. Finally, we analysed a cohort of 19 patients with CRCs who underwent unilateral or bilateral oophorectomies for ovarian metastases from CRC (Figure 1).

All patients underwent bilateral or unilateral oophorectomies via transabdominal laparotomy, with or without the resection of primary CRC or other extra-ovarian metastases and received chemotherapeutic regimens, including modified FOLFOX6 (folinic acid, fluorouracil and oxaliplatin), FOLFIRI (folinic acid, fluorouracil and irinotecan), capecitabine, CPT-11 (irinotecan, camptosar and camptothecin-11), with or without bevacizumab, cetuximab or panitumumab, either before or after surgery, at the discretion of the surgeon or the preference of the patient.

OS was calculated from the date of initiation of treatment for a primary tumour (i.e., via surgical resection or systemic chemotherapy) to the date of death or last follow-up (for censored patients). Ovarian-specific OS was calculated from the date of the first oophorectomy to the date of death or last follow-up. Ovarian-specific PFS was calculated from the date of the first oophorectomy to the date of the first documentation of local, regional, or distant relapse or the appearance of a second primary lesion, as determined by routine computed tomography scans, magnetic resonance imaging scans, or both, performed every 2–3 months.

Response criteria were defined according to the Response Evaluation Criteria in Solid Tumors (RECIST) guidelines, version 1.1 [30], as follows: CR, disappearance of all target lesions; PR, a ≥ 30% decrease in the sum of the target lesion diameters relative to the sum of baseline diameters; PD, a ≥ 20% increase in the sum of the target lesion diameters relative to the smallest sum of the preoperative tumour sites and SD, neither sufficient shrinkage of lesions to qualify for PR nor sufficient increase to qualify for PD. Pathology reports for all patients were reviewed by at least two pathologists. Institutional review board approval was granted by the ethics committee of the Okayama University, and all patients provided written informed consents for the use of their body tissues for research.

### The detection of BRAF exon 15 and KRAS exon 2 mutations

To identify mutations in *BRAF* exon 15, including in codon 600, and in *KRAS* exon 2 in each case, Sanger sequencing was performed on purified DNA isolated from formalin-fixed, paraffin-embedded, or fresh-frozen tissues from the primary tumours and corresponding ovarian metastases. The specific primer sequences for *BRAF* exon 15 and *KRAS* exon 2, and the polymerase chain reaction (PCR) conditions used here were described previously [31]. The PCR products were purified using QIAquick PCR purification kit (Qiagen, Venlo, Netherlands), and were directly sequenced using an ABI PRISM^®^ 3100-Avantä Genetic Analyzer (Applied Biosystems, Foster City, CA, USA). Examples of *BRAF* and *KRAS* mutations are shown in Supplementary Figure 3.

### Microsatellite instability analysis

All primary CRC and ovarian metastatic tissues were subjected to an MSI status analysis using four-mononucleotide repeat loci (BAT26, NR21, NR27 and CAT25), as described previously, in part [32]. Tumours that exhibited MSI in at least one mononucleotide repeat marker were classified as having MSI phenotypes, whereas those without MSI in any marker were classified as non-MSI phenotypes. An example of the MSI analysis is shown in Supplementary Figure 1.

### Statistical analyses

Statistical analyses were performed using JMP software version 10.0.2 (SAS Institute, Inc., Cary, NC, USA). Categorical variables were compared using the Chi-square test. Ovarian-specific OS and PFS were estimated by univariate analyses using the Kaplan–Meier method. Cox proportional hazard regression method was used to perform univariate and multivariate analyses of OS. The regression model included clinicopathologic factors, such as age; primary tumour location; stage per the UICC guidelines; histological and ovarian metastasis status and somatic mutation profiles, such as *BRAF or KRAS* mutations and MSI status. Spearman’s rank correlation coefficient (*ρ*) was used to analyse the relationship between ovarian-specific OS and the variables of the first oophorectomy, i.e. between the time from diagnosis of ovarian metastasis to oophorectomy, and between oophorectomy-specific OS and the rate of decrease in the number of extra-ovarian sites during chemotherapy. All reported *P* values were calculated using two-sided tests and values of < 0.05 were considered statistically significant.

## SUPPLEMENTARY MATERIALS FIGURES AND TABLES




