# Supplementary material for: Clinical outcomes of women with ovarian metastases of colorectal cancer treated with oophorectomy with respect to their somatic mutation profiles
Source: Oncotarget. 2018 Mar 27;9(23):16477–88. doi: 10.18632/oncotarget.24735 (PMC5893255; doi:10.18632/oncotarget.24735)
Supplement: Supplementary file 1 [file oncotarget-09-16477-s001.pdf]

# Clinical outcomes of women with ovarian metastases of colorectal cancer treated with oophorectomy with respect to their somatic mutation profiles

## SUPPLEMENTARY MATERIALS

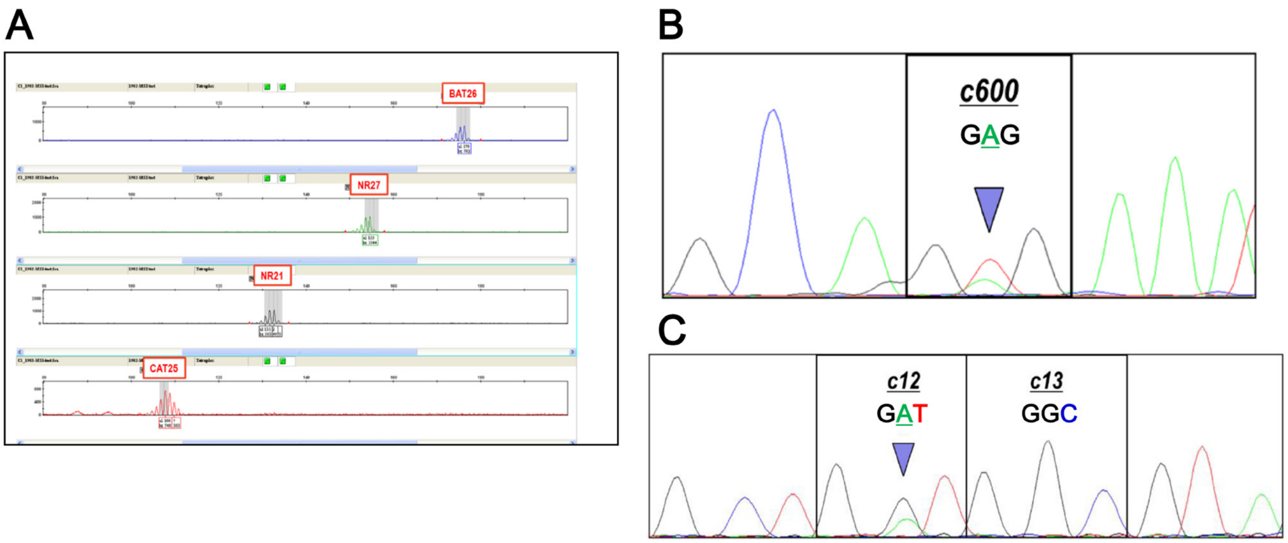


**Supplementary Figure 1:** (**A**) Tumours showing no shift in any of the four mononucleotide markers is classified as non-MSI. (**B**) An example of BRAF mutation. : The result of direct sequencing of BRAF codons 600 reveals the tumour has a BRAF V600E mutation (GTG to CAG). (**C**) The result of direct sequencing of *KRAS* codons 12 and 13 reveals the tumour has a *KRAS* codon 12 mutation (GGT to GAT).

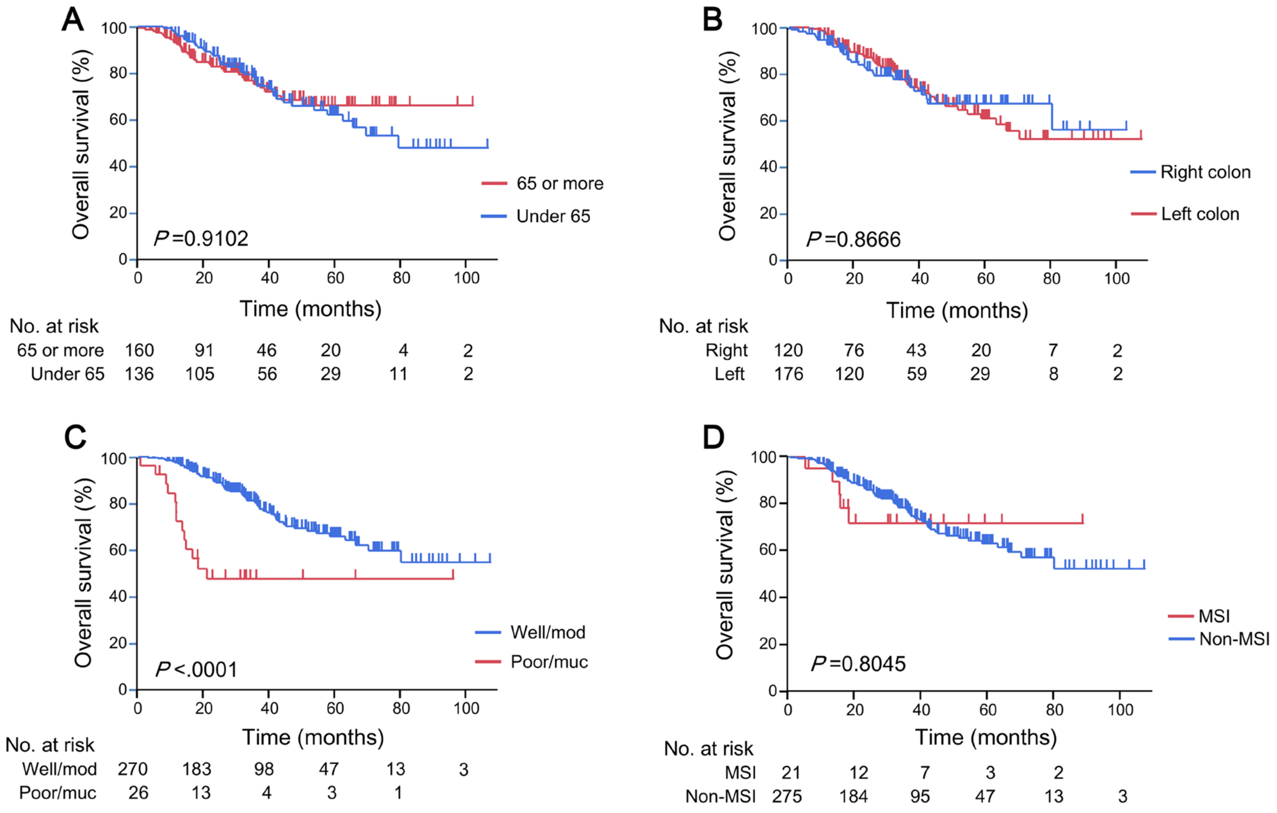


**Supplementary Figure 2:** Kaplan-Meier curves for 296 female CRC patients were estimated by (**A**) age, (**B**) location of the primary tumour, (**C**) histology, and (**D**) MSI status. *P* values were calculated by the log-rank test. Overall survival was estimated after initial therapies. Well, well differentiated adenocarcinoma; mod, moderately differentiated adenocarcinoma; poor/muc, poor differentiated or mucinous adenocarcinoma; MSI, microsatellite instability.

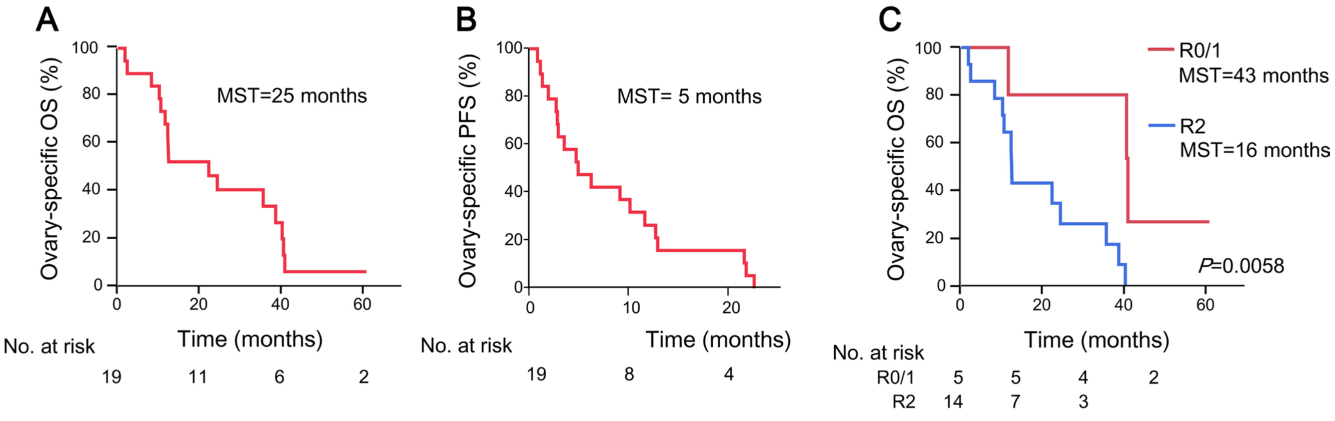


**Supplementary Figure 3:** (**A**) Kaplan–Meier estimates of ovary-specific OS; (**B**) Kaplan-Meier curves for ovary-specific PFS in 19 female CRC patients with ovary metastases. (**C**) Kaplan–Meier curves for ovary-specific OS by curability. A *P* value was calculated by the log-rank test.

**Supplementary Table 1: Patients' characteristics, genetic status, time to oophorectomy, and outcome.** See Supplementary_Table 1

## Supplementary Table 2: Response to chemotherapy

| Patient case no. | Surgery | Preoperative chemotherapy | Responses in ovary | Responses at extraovarian sites | Oophorectomy-specific PFS (M) | Oophorectomy-specific OS (M) | Outcome |
|---|---|---|---|---|---|---|---|
| 1 | secondary unilateral oophorectomy | + | PD | SD | 1 | 10 | DOD |
| 2 | primary bilateral oophorectomy | + | PD | CR | 13 | 42 | DOD |
| 3 | primary bilateral oophorectomy | + | SD | no extraovarian metastasis | 2 | 41 | DOD |
| 4 | primary unilateral oophorectomy | + | PD | PR | 12 | 40 | DOD |
| 5 | primary bilateral oophorectomy | + | SD | PR | 5 | 38 | DOD |
| 6 | secondary unilateral oophorectomy | + | PD | PD | 2 | 13 | DOD |
| 8 | primary unilateral oophorectomy | + | PD | PR | 3 | 14 | DOD |
| 9 | primary unilateral oophorectomy | + | PD | PR | 3 | 7 | DOD |
| 12 | primary bilateral oophorectomy | + | PD | SD | 3 | 16 | DOD |
| 17 | primary unilateral oophorectomy | + | PD | SD | 1 | 7 | DOD |

CR, complete response; PR, partial response; SD, stable disease; PD, progressive disease; PFS, progress free survival; OS, overall survival; DOD, Dead of disease.
