# Supplementary material for: Clinical outcomes of women with ovarian metastases of colorectal cancer treated with oophorectomy with respect to their somatic mutation profiles
Source: Oncotarget. 2018 Mar 27;9(23):16477–88. doi: 10.18632/oncotarget.24735 (PMC5893255; doi:10.18632/oncotarget.24735)
Supplement: Supplementary file 2 [file oncotarget-09-16477-s002.docx]

**Supplementary Table 1: Patients’ characteristics, genetic status, time to oophorectomy, and outcome**

| Patient no | Time in relation to primary tumours | Primary colorectal cancer site | *BRAF/KRAS* mutation status; Primary site/Ovarian metastasis site | MSI status; Primary site/Ovarian metastasis site | Age | Stage at diagnosis | Other metastasis | First oophorectomy | Duration of diagnosis for ovarian metastasis from the start of the CRC treatment | Duration of first oophorectomy from diagnosis of ovarian metastasis | Duration of diagnosis for residual ovarian metastasis from the first oophorectomy | CEA (ng/mL) | Size of ovarian metastases (mm) | OSS (M) | OS (M) | OS (M) after initial therapy to stage IV or recurrence | Outcome |
| --- | --- | --- | --- | --- | --- | --- | --- | --- | --- | --- | --- | --- | --- | --- | --- | --- | --- |
| 1 | Antechronous | Rectum | Wild-type/ Wild-type | Non-MSI/ Non-MSI | 41 | IVB | None | Hemi | -5 | -5 | 4 | Unknown | 160 | 16 | 16 | 16 | DOD |
| 2 | Metachronous | Transverse colon | Wild-type/ Wild-type | Non-MSI/ Non-MSI | 64 | IVB | Dissemination | Bil. | 38 | 15 |  | 27.7 | 156 | 42 | 80 | 42 | DOD |
| 3 | Metachronous | Sigmoid colon | *KRAS* G12A / *KRAS* G12A | Non-MSI/ Non-MSI | 48 | IIIC | None | Bil. | 24 | 2 |  | 15.2 | 32 | 43 | 66 | 49 | DOD |
| 4 | Metachronous | Sigmoid colon | Wild-type/ Wild-type | Non-MSI/ Non-MSI | 52 | IVA | Liver | Hemi | 14 | 2 |  | 61.1 | 189 | 40 | 54 | 54 | DOD |
| 5 | Metachronous | Rectosigmoid colon | Wild-type/ *KRAS* G12S | Non-MSI/ Non-MSI | 54 | IIIA | Dissemination  LN | Bil. | 26 | 3 |  | 1 | 62 | 38 | 63 | 40 | DOD |
| 6 | Metachronous | Sigmoid colon | Wild-type/ Wild-type | Non-MSI/ Non-MSI | 38 | IVA | None | Hemi | 10 | 1 | 12.7 | Unknown | Unknown | 27 | 37 | 37 | DOD |
| 7 | Metachronous | Cecum | Wild-type/ *KRAS* G12V | Non-MSI/ Non-MSI | 69 | IVA | Liver | Bil. | 22 | 1 |  | Unknown | 156 | 16 | 38 | 38 | DOD |
| 8 | Metachronous | Sigmoid colon | Wild-type/ Wild-type | Non-MSI/ Non-MSI | 59 | IVB | Lung  Liver | Hemi | 21 | 15 |  | 7 | 90 | 14 | 36 | 36 | DOD |
| 9 | Metachronous | Ascending colon | *BRAF* V600E*/ BRAF* V600E | Non-MSI/ Non-MSI | 54 | IVB | Dissemination  Bone | Hemi | 15 | 12 |  | 558.1 | 207 | 7 | 21 | 21 | DOD |
| 10 | Synchronous | Sigmoid colon | Wild-type/ Wild-type | Non-MSI/ Non-MSI | 35 | IVA | Dissemination  Liver | Hemi | 0 | 0 | 35.4 | Unknown | 140 | 61 | 61 | 61 | Alive |
| 11 | Synchronous | Ascending colon | *KRAS* G12V/ *KRAS* G12V | Non-MSI/ Non-MSI | 80 | IVB | None | Bil. | 0 | 1 |  | 11 | 116 | 29 | 29 | 29 | Alive |
| 12 | Metachronous | Rectum | *BRAF* V600E*/ BRAF* V600E | Non-MSI/ Non-MSI | 63 | IIIB | Dissemination  Lung | Bil. | 16 | 6 |  | 18.6 | 132 | 16 | 32 | 22 | DOD |
| 13 | Synchronous | Sigmoid colon | Wild-type/ Wild-type | Non-MSI/ Non-MSI | 65 | IVB | Liver  LN | Hemi | 0 | 0 |  | 53.3 | Unknown | 15 | 15 | 15 | DOD |
| 14 | Synchronous | Sigmoid colon | Wild-type/ NA | Non-MSI/ NA | 46 | IVB | Dissemination | Bil. | 0 | 1 |  | Unknown | Unknown | 25 | 25 | 25 | DOD |
| 15 | Synchronous | Transverse colon | *KRAS* G12V/ *KRAS* G12V | Non-MSI/ Non-MSI | 44 | IVB | None | Bil. | 0 | 0 |  | 676.2 | Unknown | 24 | 24 | 24 | Alive |
| 16 | Metachronous | Ascending colon | Wild-type/ Wild-type | Non-MSI/ Non-MSI | 56 | IVB | Dissemination  Liver | Bil. | 8 | 0 |  | 12.1 | Unknown | 16 | 24 | 24 | DOD |
| 17 | Synchronous | Transverse colon | *KRAS* G12V/ *KRAS* G12V | Non-MSI/ Non-MSI | 34 | IVB | Liver  Lung  LN | Hemi | 1 | 2 |  | 30.09 | 30 | 7 | 9 | 9 | DOD |
| 18 | Metachronous | Ascending colon | *BRAF* V600E*/ BRAF* V600E | Non-MSI/ Non-MSI | 39 | IIIC | Dissemination | Bil. | 19 | 0 |  | 1.6 | Unknown | 13 | 31 | 25 | DOD |
| 19 | Synchronous | Cecum | *KRAS* G12D/ Wild-type | Non-MSI/ Non-MSI | 58 | IVB | Dissemination | Bil. | 0 | -1 |  | 13.2 | 140 | 41 | 41 | 41 | DOD |

*Antechronous, primary colorectal cancer discovered after oophorectomy; MSI, microsatellite instability positive; Age, age of diagnosis of the first ovarian metastasis; LN, lynph node metastasis; Hemi, hemi-oophorectomy; Bil., bilateral oophorectomy; OSS, Ovary-specific survival from the first oohorectomy; OS, overall survival after initial treatment; DOD, dead of diasese
